# One Step Closer to Clinical Translation: Enhanced Tumor Targeting of [^99m^Tc]Tc-DB4 and [^111^In]In-SG4 in Mice Treated with Entresto

**DOI:** 10.3390/pharmaceutics12121145

**Published:** 2020-11-26

**Authors:** Panagiotis Kanellopoulos, Aikaterini Kaloudi, Maritina Rouchota, George Loudos, Marion de Jong, Eric P. Krenning, Berthold A. Nock, Theodosia Maina

**Affiliations:** 1 Molecular Radiopharmacy, INRASTES, NCSR “Demokritos”, 15341 Athens, Greece; kanelospan@gmail.com (P.K.); katerinakaloudi@yahoo.gr (A.K.); nock_berthold.a@hotmail.com (B.A.N.); 2 Molecular Pharmacology, School of Medicine, University of Crete, 70013 Heraklion, Greece; 3 BIOEMTECH, Lefkippos Attica Technology Park NCSR “Demokritos”, 15310 Athens, Greece; mrouchota@bioemtech.com (M.R.); george@bioemtech.com (G.L.); 4 Department of Radiology & Nuclear Medicine Erasmus MC, 3015 CN Rotterdam, The Netherlands; m.hendriks-dejong@erasmusmc.nl; 5 Cyclotron Rotterdam BV, Erasmus MC, 3015 CE Rotterdam, The Netherlands; erickrenning@gmail.com

**Keywords:** tumor-targeting, peptide radioligand, [^99m^Tc]Tc-DB4, gastrin-releasing peptide receptor, [^111^In]In-SG4, cholecystokinin subtype 2 receptor, single photon emission computed tomography, neprilysin inhibition, Entresto, LBQ657, in vivo stability

## Abstract

Background: Peptide radioligands may serve as radionuclide carriers to tumor sites overexpressing their cognate receptor for diagnostic or therapeutic purposes. Treatment of mice with the neprilysin (NEP)-inhibitor phosphoramidon was previously shown to improve the metabolic stability and tumor uptake of biodegradable radiopeptides. Aiming to clinical translation of this methodology, we herein investigated the impact of the approved pill Entresto, releasing the potent NEP-inhibitor LBQ657 in vivo, on the stability and tumor uptake of two radiopeptides. Methods: The metabolic stability of [^99m^Tc]Tc-DB4 (DB4, N_4_-Pro-Gln-Arg-Tyr-Gly-Asn-Gln-Trp-Ala-Val-Gly-His-Leu-Nle-NH_2_) and [^111^In]In-SG4 (SG4, DOTA-DGlu-Ala-Tyr-Gly-Trp-Nle-Asp-Phe-NH_2_) was tested in LBQ657/Entresto-treated mice vs. untreated controls. The uptake in gastrin-releasing peptide receptor (GRPR)-, or cholecystokinin subtype 2 receptor (CCK_2_R)-positive tumors respectively, was compared between LBQ657/Entresto-treated mice and untreated controls. Results: LBQ657/Entresto treatment induced marked stabilization of [^99m^Tc] Tc-DB4 and [^111^In]In-SG4 in peripheral mice blood, resulting in equally enhanced tumor uptake at 4 h post-injection. Accordingly, the [^99m^Tc]Tc-DB4 uptake of 7.13 ± 1.76%IA/g in PC-3 tumors increased to 16.17 ± 0.71/17.50 ± 3.70%IA/g (LBQ657/Entresto) and the [^111^In]In-SG4 uptake of 3.07 ± 0.87%IA/g in A431-CCK_2_R(+) tumors to 8.11 ± 1.45/9.61 ± 1.70%IA/g. Findings were visualized by SPECT/CT. Conclusions: This study has shown the efficacy of Entresto to notably improve the profile of [^99m^Tc]Tc-DB4 and [^111^In]In-SG4 in mice, paving the way for clinical translation of this approach.

## 1. Introduction

Latest advances in medicine have been aiming at personalized management of patients. Toward this goal, modern anti-cancer drugs are being designed to specifically interact with tumor-associated biomolecular targets, sparing healthy tissues devoid of target expression. For example, peptide analogs may serve as radionuclide carriers to tumor-located receptors, allowing for diagnostic imaging and/or radionuclide cancer therapy—“cancer theranostics” [1,2,3,4]. Diagnostic imaging may be performed by means of gamma emitters (e.g., technetium-99m, indium-111) applying single photon emission tomography (SPECT) or via positron emitters (e.g., fluorine-18, gallium-68, copper-64) in combination with positron emission tomography (PET). On the other hand, particle emitters (e.g., lutetium-177, copper-67, actinium-225) accumulating on tumor sites by suitably designed carriers will locally deliver cytotoxic payloads, eradicating tumor lesions [5,6,7]. This approach has been successfully implemented in the clinic for neuroendocrine tumor patients with the advent of theranostic somatostatin-based radioligands, such as the [^68^Ga]Ga-/[^177^Lu]Lu-DOTA-TATE pair (DOTA-DPhe-c[Cys-Tyr-DTrp-Lys-Thr-Cys]-Thr-OH, DOTA = 1,4,7,10-tetraazacyclododecane-1,4,7,10-tetraacetic acid) [8].

A major hurdle for a broader implementation of this method in nuclear oncology relates to the notorious propensity of peptides to metabolic degradation. Enzymes hydrolyzing peptide bonds, peptidases, rapidly catabolize radiopeptides entering the circulation, impairing radionuclide delivery and uptake in pathological lesions. A typical way to increase the resistance of radiopeptides to assaulting peptidases is through structural modifications of the peptide chain, such as amino acid replacements, cyclization, reduction or methylation of peptide bonds and other means, which, however, often negatively affect other important biological features, such as receptor affinity and pharmacokinetics [9,10,11].

We have previously shown that the in vivo profile of linear radiopeptides originating from either the bombesin (BBN, Pyr-Gln-Arg-Leu-Gly-Asn-Gln-Trp-Ala-Val-Gly-His-Leu-Met-NH_2_), or the minigastrin (MG, H-Leu-Glu-Glu-Glu-Glu-Glu-Ala-Tyr-Gly-Trp-Met-Asp-Phe-NH_2_) peptide families, and targeting the gastrin-releasing peptide receptor (GRPR) or the cholecystokinin subtype 2 receptor (CCK_2_R) respectively, on tumors, are compromised primarily by the action of a single peptidase, neprilysin (NEP) [12,13,14]. Most interestingly, we were able to show that co-injection of the NEP-inhibitor phosphoramidon (PA) [15,16] induced significant stabilization of radiopeptides in peripheral blood, thereby markedly enhancing tumor uptake in mice models [12,17,18,19,20,21]. This simple but effective concept warrants validation in the clinic in view of the prospects it offers for a broader and more effective application of radiopeptides in cancer theranostics. For such purposes, the availability of NEP-inhibitors which are registered medicines and currently in clinical use is of great value [21].

**Figure 1:**
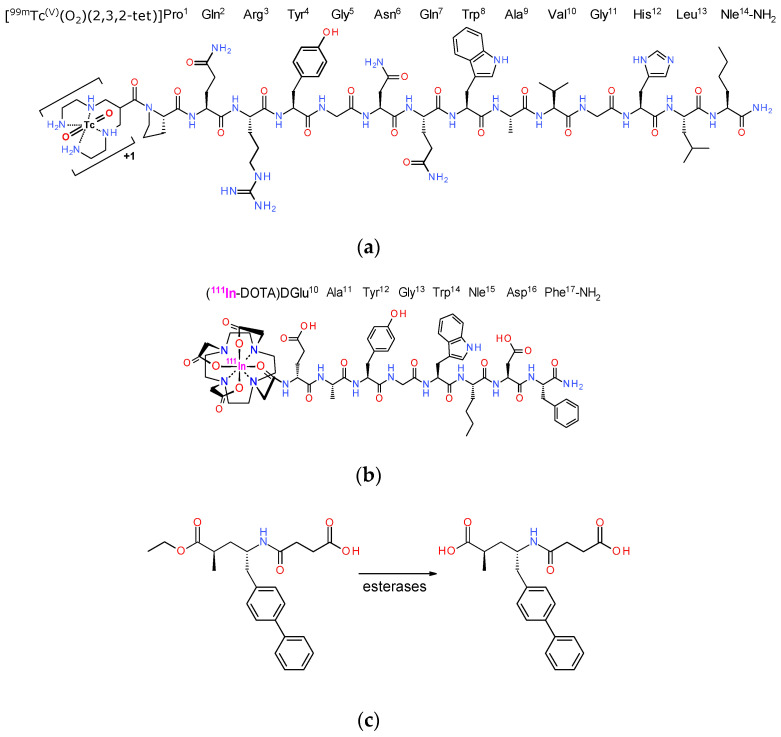
Structure of (**a**) [^99m^Tc]Tc-DB4, (**b**) [^111^In]In-SG4 and (**c**) sacubitril (AHU377) contained in Entresto pills and releasing the active substance sacubitrilat (LBQ657) in vivo upon ester-hydrolysis by esterases. Sacubitrilat is a potent and specific inhibitor of neprilysin (NEP).

In the present work, we decided to study the effects of such an approved NEP-inhibitor firstly on the metabolic stability of two representative radiopeptide examples, (i) the BBN-based [^99m^Tc]Tc-DB4 (DB4, N_4_-Pro-Gln-Arg-Tyr-Gly-Asn-Gln-Trp-Ala-Val-Gly-His-Leu-Nle-NH_2_) [22,23,24] and (ii) [^111^In]In-SG4 (SG4, DOTA-DGlu-Ala-Tyr-Gly-Trp-Nle-Asp-Phe-NH_2_) [20,24], generated from truncated MG (Figure 1a,b). It should be noted that both [^99m^Tc]Tc-DB4 [23] and [^111^In]In-MG11 (a Met^11^-derivative of [^111^In]In-SG4) have been previously clinically tested in a small number of prostate cancer and medullary thyroid carcinoma patients [20,21,24,25]. As an approved NEP-inhibitor, we selected sacubitrilat (LBQ657), which we administered in mice either intravenously together with the radiopeptide, or per os via the registered pill Entresto, 30 min prior to radiopeptide injection, closely mimicking a clinical setting. Entresto pills contain the LBQ657-precursor, sacubitril (AHU377), and release the active substance LBQ657 after ester-hydrolysis by in vivo esterases (Figure 1c) [26,27,28,29]. Next, we compared the impact of treating the animals with either LBQ657 or Entresto vs. non-treated control animals bearing subcutaneous tumors. In particular, for [^99m^Tc]Tc-DB4, these treatments were compared in mice bearing GRPR-positive human prostate adenocarcinoma PC-3 xenografts [30] and for [^111^In]In-SG4, in mice bearing a double A431-CCK_2_R((+/−)) tumor model [31]. Conclusions on the feasibility and efficacy of improving the tumor-targeting and pharmacokinetics of these two representative radiopeptides with the aid of registered Entresto pills were drawn.

## 2. Materials and Methods

### 2.1. Peptide Analogs—Inhibitors—Radioligands

#### 2.1.1. Chemicals—Radionuclides

The DB4 (N_4_-Pro-Gln-Arg-Tyr-Gly-Asn-Gln-Trp-Ala-Val-Gly-His-Leu-Nle-NH_2_, N_4_ = 6-(carboxy)-1,4,8,11-tetraazaundecane) and SG4 (DOTA-DGlu-Ala-Tyr-Gly-Trp-Nle-Asp-Phe-NH_2_, DOTA = 1,4,7,10-tetraazacyclododecane-1,4,7,10-tetraacetic acid) peptide conjugates (Figure 1) were provided by PiChem (Graz, Austria). [Tyr^4^]BBN (Pyr-Gln-Arg-Tyr-Gly-Asn-Gln-Trp-Ala-Val-Gly-His-Leu-Met-NH_2_) was purchased from Bachem (Bubendorf, Switzerland). The neprilysin (NEP)-inhibitor sacubitrilat (LBQ657, (2*R*,4*S*)-4-(3-carboxypropanoylamino)-2-methyl-5-(4-phenylphenyl)-pentanoic acid) was obtained from BIOMOL GmbH (Hamburg, Germany), while Entresto pills (200 mg corresponding to 24 mg/26 mg sacubitril/valsartan per pill) containing the prodrug sacubitril (AHU377, 4-[[(2*S*,4*R*)-5-ethoxy-4-methyl-5-oxo-1-(4-phenylphenyl)pentan-2-yl]amino]-4oxobutanoic acid) (Figure 1) (Novartis, Basel, Switzerland) were purchased from a local pharmacy. For animal treatment (vide infra), whole pills were ground to a fine powder in a mortar, divided and suspended in tab water to individual 12 mg/200 mL doses of pill per animal [32].

Technetium-99m in the form of [^99m^Tc]NaTcO_4_ was collected by elution of a [^99^Mo]Mo/[^99m^Tc]Tc generator (Ultra-Technekow™ V4 Generator, Curium Pharma, Petten, The Netherlands). Indium-111 as an [^111^In]InCl_3_ solution was purchased from Curium Pharma (Petten, The Netherlands).

#### 2.1.2. Preparation of Radioligands

Lyophilized DB4 and SG4 were dissolved in HPLC-grade H_2_O to yield a 1 mM stock solution and 50 μL aliquots thereof were stored in Eppendorf Protein LoBind tubes at −20 °C.

Labeling of DB4 with technetium-99m was conducted in molar activities of 18–37 MBq/nmol in an Eppendorf protein LoBind tube, wherein the following solutions were consecutively added: (i) 0.5 M phosphate buffer pH 11.5 (50 µL), (ii) 0.1 M sodium citrate (5 µL), (iii) [^99m^Tc]NaTcO_4_ generator eluate (415 mL, 280–550 MBq), (iv) DB4 stock solution (15 µL, 15 nmol) and (v) freshly made SnCl_2_ solution in EtOH (15 µg, 15 µL). After reaction for 30 min at ambient temperature, the pH was brought to ~7 by adding 0.1 M HCl.

SG4 was labeled with indium-111 in molar activities of 3.7–7.4 MBq/nmol. Briefly, [^111^In]InCl_3_ (30 µL, in 50 mM HCl; corresponding to 11.1–22.2 MBq) was added to an Eppendorf protein LoBind tube followed by the addition of SG4 (3 µL, 3 nmol) stock solution and 1 M sodium acetate buffer (3 µL, pH 4.6). The labeling reaction mixture was incubated for 30 min at 85 °C. For quality control, a 2 µL aliquot of the labeling solution was withdrawn and quenched with 28 µL of an acetate buffered solution of diethylenetriaminepentaacetic acid (DTPA, 1 mM, pH 4.6). Finally, traces of free indium-111 in the labelling solution were scavenged by addition of DTPA to a 1 mM final concentration. For small-animal SPECT studies, the labelling reaction was performed with 150 μL of [^111^In]InCl_3_ solution (55–110 MBq) and [^111^In]In-SG4 was isolated using a Symmetry Shield RP18 cartridge column (5 μm, 3.9 × 20 mm, Waters, Eschborn, Germany). The column was eluted for 40 min with a linear gradient from 100% A (15 mM ammonium acetate )/0% B (acetonitrile, MeCN) to 90% A/10% B in 5 min, then in 10 min to 85% A/15% B, and afterwards, isocratic at 1 mL/min flow rate. [^111^In]In-SG4, *t*_R_ = 28.4 min; SG4, *t*_R_ = 25.5 min; [^111^In]In-DTPA, *t*_R_ = 1.1 min.

#### 2.1.3. Quality Control

Reversed-phase high-performance liquid chromatography (RP-HPLC) was performed on a Waters Chromatograph based on a 600E multi-solvent delivery system coupled to a Waters 2998 photodiode array detector (Waters, Vienna, Austria) and a Gabi gamma-detector (Raytest, RSM Analytische Instrumente GmbH, Straubenhardt, Germany). Data processing and chromatography were controlled by the Empower Software (Waters, Milford, MA, USA). For quality control, aliquots of the radiolabeling solution were loaded on a Symmetry Shield RP18 cartridge column (5 μm, 3.9 × 20 mm, Waters, Eschborn, Germany), eluted for 40 min with 0.1% trifluoroacetic acid (TFA) in H_2_O/MeCN applying a linear gradient starting from 0% MeCN and a 2% increase per min at 1 mL/min flow rate (system 1). Instant thin-layer chromatography (ITLC) analysis was additionally performed for [^99m^Tc]Tc-DB4 on ITLC-SG strips (Pall Corporation, Port Washington, NY/USA), developed up to 10 cm from the origin with 5 M ammonium acetate/MeOH 1/1 (*v*/*v*) for [^99m^Tc]TcO_2_ × nH_2_O (R_f_: 0; [^99m^Tc]Tc-DB4 R_f_: 0.8–1.0; [^99m^Tc]TcO_4_^−^ R_f_: 0.8–1.0), or acetone for [^99m^Tc]TcO_4_^−^ detection (R_f_: 1; [^99m^Tc]Tc-DB4 and [^99m^Tc]TcO_2_ × nH_2_O R_f_: 0). The radiochemical labeling yields exceeded 98% and the radiochemical purity was >99%, and thus radioligands were used without further purification in all subsequent experiments, with the exception of the SPECT/CT imaging experiment. Furthermore, samples of [^99m^Tc]Tc-DB4 and [^111^In]In-SG4 were tested before and after the end of all biological experiments to monitor radiotracer integrity.

All manipulations with beta^−^- and gamma-emitting radionuclides and their solutions were performed by trained and authorized personnel behind suitable shielding in licensed laboratories in compliance with European radiation-safety guidelines and supervised by the Greek Atomic Energy Commission (license # A/435/17092/2019).

### 2.2. Biological Assays

#### 2.2.1. Cell Culture

Human androgen-independent prostate adenocarcinoma PC-3 cells endogenously expressing the GRPR [30] were obtained from LGC Standards GmbH (Wesel, Germany), whereas the human epidermoid carcinoma A431 cell line transfected to stably express the human CCK_2_R (A431-CCK_2_R(+)) [31,33] or devoid of CCK_2_R expression (A431-CCK_2_R(‒)) was a gift from Prof. O. Boerman (Department of Nuclear Medicine, Radboud University Nijmegen Medical Centre, Nijmegen, The Netherlands) and Prof. L. Aloj (Istituto di Biostrutture e Bioimmagini, Consiglio Nazionale delle Ricerche, Naples, Italy). All culture reagents were obtained from Gibco BRL, Life Technologies (Grand Island, NY, USA) or from Biochrom KG Seromed (Berlin, Germany).

Roswell Park Memorial Institute-1640 (RPMI-1640) medium with GlutaMAX-I and supplemented with 10% (*v*/*v*) fetal bovine serum (FBS), 100 U/mL penicillin and 100 μg/mL streptomycin was used for PC-3 cell culture. A431-CCK_2_R(+/−) cells were grown in Dulbecco’s Modified Eagle medium (DMEM) with GlutaMAX-I, supplemented with 10% (*v*/*v*) fetal bovine serum, 100 U/mL penicillin and 100 μg/mL streptomycin. In the case of A431-CCK_2_R(+) cells, the medium was additionally supplemented with 250 μg/mL G418. Cells were kept in a controlled humidified air containing 5% CO_2_ at 37 °C. Splitting of cells with a ratio of 1:2 to 1:5 was performed when approaching confluency using a trypsin/ethylenediaminetetraacetic acid (EDTA) solution (0.05%/0.02% *w*/*v*).

#### 2.2.2. Animal Studies

European guidelines were applied in all animal studies in supervised and licensed facilities (EL 25 BIO 021). The study protocols were approved by the Department of Agriculture and Veterinary Service of the Prefecture of Athens (revised protocol number 1609 approved on 24 April 2019 for the stability studies and revised protocol number 1610 approved on 24 April 2019 for biodistribution and imaging studies). For stability experiments, 20 healthy male Swiss albino mice (30 ± 5 g, NCSR “Demokritos” Animal House Facility) were used, divided in groups of 3 or 4 (vide infra). For biodistribution studies with [^99m^Tc]Tc-DB4, 16 female severe combined immune deficiency (SCID) mice (14.3 ± 1.3 g, six weeks of age at the day of arrival, NCSR “Demokritos” Animal House Facility) were employed, divided in 4 groups of 4. Biodistribution and imaging studies with [^111^In]In-SG4 were conducted in 17 male SCID mice (17.2 ± 3.0 g, six weeks of age at the day of arrival) from the same provider. 12 animals divided in 3 groups of 4 were used in biodistribution studies and 5 in SPECT/CT imaging.

#### 2.2.3. In Vivo Stability Tests

A 100 μL bolus of [^99m^Tc]Tc-DB4 (50–60 MBq, 3 nmol total peptide in vehicle: saline/EtOH 9/1 *v*/*v*) or [^111^In]In-SG4 (11–22 MBq, 3 nmol of total peptide in saline/EtOH 9/1 *v*/*v*) was injected in the tail vein of mice together with vehicle (100 µL; control; [^99m^Tc]Tc-DB4, *n* = 3 or [^111^In]In-SG4, *n* = 4) or with LBQ657 solution (100 µL injection solution containing 10 µg LBQ657, *n* = 3 for both analogs). In a third set of animals per radioligand, mice received, by gavage, a single Entresto dose (12 mg/200 mL per animal, prepared as described in Section 2.1.1.) 30 min prior to injection of either radiotracer together with vehicle (100 μL; Entresto-group; [^99m^Tc]Tc-DB4, *n* = 4 or [^111^In]In-SG4, *n* = 3). Mice were euthanized 5 min after radioligand injection (5 min post-injection (pi)) and a prechilled syringe was used to withdraw blood (0.5–1 mL) directly from the heart. Blood was transferred in a pre-chilled EDTA-containing Eppendorf tube on ice and subsequently centrifuged for 10 min at 2000 *g*/4 °C. The plasma was collected and an equal volume of ice-cold MeCN was added. The mixture was centrifuged for 10 min at 15,000 *g*/4 °C. The supernatant was collected and a N_2_-flux at 40 °C was applied to reduce the volume to 0.05–0.1 mL. After diluting with normal saline (0.4 mL), the solution was passed through a 0.22 μm Millex GV filter (Millipore, Milford, USA). Filtrate aliquots were then analyzed by radio-HPLC. A Symmetry Shield RP18 (5 μm, 3.9 × 20 mm) column served as stationary phase, eluted at a flow rate of 1 mL/min by 0.1% TFA in H_2_O (A) and MeCN (B) combinations as mobile phase. In case of [^99m^Tc]Tc-DB4, the following linear gradient system was applied: 0%B at 0 min, 10%B at 10 min and reaching 25%B at 40 min (system 2a); for [^111^In]In-SG4, the gradient started with 0%B increasing linearly with 1%/min to 40%B (system 2b). The *t*_R_ of the intact radiopeptide was determined by co-injection with the respective [^99m^Tc]Tc-DB4 (*t*_R_ = 31.5 min)/[^111^In]In-SG4 (*t*_R_ = 30.0 min) reference in the HPLC.

#### 2.2.4. Tumor Induction in Mice

Aliquots of a suspension containing freshly harvested human PC-3 cells (≈1.2 × 10^7^ cells in 150 µL normal saline per mouse) were inoculated at the flanks of 16 female SCID mice and 3 weeks later, well-palpable tumors (124 ± 35 mg) were grown at the inoculation sites. Inocula of A431-CCK_2_R(+) and A431-CCK_2_R(−) cells (1.5 × 10^7^ cells and 1.2 × 10^7^ cells respectively, in 150 µL normal saline) were subcutaneously injected in the right and left flanks respectively, of 12 male SCID mice; in 5 more mice, a A431-CCK_2_R(+) cell suspension (1.5 × 10^7^ cells in 150 µL normal saline) was subcutaneously injected instead on the neck of the animals. Well-palpable tumors were developed in all cases after 10 days (A431-CCK_2_R(+): 188 ± 56 mg; A431-CCK_2_R(−): 124 ± 49 mg). All animals were kept under aseptic conditions during this period and until biodistribution or imaging was conducted.

#### 2.2.5. Biodistribution of [^99m^Tc]Tc-DB4 and [^111^In]In-SG4 in Tumor-Bearing Mice

For the biodistribution study, animals in groups of 4 received a 100 μL bolus of [^99m^Tc]Tc-DB4 via the tail vein (200 kBq, corresponding to 10 pmol total peptide in saline/EtOH 9/1 *v/v—*GRPR-tumor-bearing mice) or [^111^In]In-SG4 (50 kBq, 10 pmol total peptide—twin A431-CCK_2_R(+)/A431-CCK_2_R(−) tumor-bearing mice) co-injected either with injection solution (100 μL; control) or with LBQ657 solution (100 µL injection solution containing 10 µg LBQ657). In additional sets of animals, mice received, by gavage, a single Entresto dose (12 mg/200 mL per animal, prepared as described in Section 2.1.1) 30 min prior to injection of the respective radiotracer together with vehicle (100 μL; Entresto group), or with a [Tyr^4^]BBN solution (100 μg in 100 μL vehicle; block group). Mice were euthanized at 4 h pi and dissected. Samples of blood, tumors and organs of interest were collected, weighed and measured for radioactivity in the γ-counter (an automated well-type gamma counter with a NaI(Tl) 3′’ crystal, Canberra Packard Auto-Gamma 5000 series instrument). Intestines and stomach were not emptied of their contents; exceptionally, in the case of [^111^In]In-SG4-injected animals, stomachs were emptied of their contents. Data was calculated as percent injected activity per gram tissue (%IA/g) with the aid of standard solutions and represented as mean values ± standard deviation (SD), *n* = 4.

#### 2.2.6. Statistical Analysis

The two-way analysis of variance (ANOVA) with multiple comparisons was used for statistical analysis of biological results, applying Tukey’s post hoc analysis (GraphPad Prism Software 6, San Diego, CA, USA). *p*-values below 0.05 were considered to be statistically significant.

#### 2.2.7. SPECT/CT Imaging of [^111^In]In-SG4 in A431-CCK_2_R(+) Tumor-Bearing Mice

Five mice bearing A431-CCK_2_R(+) xenografts on their shoulders were used for SPECT/CT imaging. Three out of five mice received Entresto by gavage 30 min prior to radiotracer injection. All five animals were injected with a bolus containing [^111^In]In-SG4 (100 μL, 5 MBq of HPLC isolated preparation) via the tail vein and were euthanized 4 h later. The y-CUBE/x-CUBE systems (Molecubes, Belgium) were employed for tomographic SPECT/CT imaging [34]. The SPECT system was based on monolithic NaI detectors attached to SiPMs, with a 0.6 mm intrinsic resolution. The CT system was based on a structured CMOS detector of CsI with pixels of 75 µm and operated between 35 and 80 kVp, 10–500 µA tube current, with a 33 µm fixed focal spot size. For SPECT scans, a 60 min duration protocol was applied, based on the injected activity. A CT scan followed after each SPECT scan, according to a General Purpose protocol under 50 kVp for co-registration purposes. The MLEM reconstruction method was applied for the reconstruction of SPECT images with a 250 µm voxel size and 500 iterations. The ISRA reconstruction method with a 100 µm voxel size was applied for the reconstruction of CT images. Images were exported and post-processed on VivoQuant software, version 4.0 (Invicro, Boston, MA, USA). A smoothing median filter (0.6 mm, spherical window) was applied to the images and for consistency purposes, the bladder was removed.

## 3. Results

### 3.1. Peptides and Radioligands

Labeling of DB4 with technetium-99m typically proceeded by brief peptide-conjugate incubation with [^99m^Tc]TcO_4_^−^ generator eluate, SnCl_2_ as reducing agent and citrate anions as transfer ligand in alkaline pH at ambient temperature at molecular activities of 18–37 MBq [^99m^Tc]Tc/nmol peptide. Labeling of SG4 with indium-111 was accomplished by 30 min incubation of the peptide-conjugate at 85 °C with [^111^In]InCl_3_ in acidic medium at molecular activities 3.7 × 7.4 MBq [^111^In]In/nmol peptide. Quality control of the radiolabeled products combined HPLC and ITLC analysis. The total radiochemical impurities in the case of [^99m^Tc]Tc-DB4, comprising [^99m^Tc]TcO_4_^−^, [^99m^Tc]Tc-citrate and [^99m^Tc]TcO_2_ nH_2_O, did not exceed 2%, while a single radiopeptide species was detected by RP-HPLC. Likewise, labeling yields above 98% and radiochemical purity above 99% were confirmed for [^111^In]In-SG4 during HPLC analysis. Therefore, the radioligands were used without further purification in all subsequent experiments, except for imaging (vide infra).

### 3.2. In Vivo Studies

#### 3.2.1. Stability of [^99m^Tc]Tc-DB4 and [^111^In]In-SG4 in Mice

**Figure 2:**
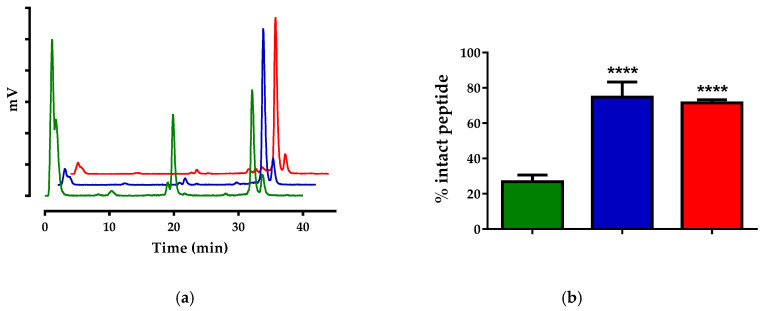
(**a**) Representative radiochromatograms of HPLC analysis (HPLC system 2a) of mice blood samples collected 5 min pi of [^99m^Tc]Tc-DB4, co-injected with vehicle (green line), LBQ657 (blue line) or co-injected with vehicle 30 min after oral administration of Entresto (red line). (**b**) Comparison of percent intact [^99m^Tc]Tc-DB4 across treatments (green, blue and red bars, respectively), showing statistically significant differences between LBQ657- or Entresto-treated mice vs. controls (**** *p* < 0.0001). Numerical average values ± standard deviation (SD) are summarized in Table 1.

Both radiotracers were catabolized to a great extent 5 min after their entry in mice circulation. As revealed by HPLC analysis of blood samples collected at 5 min pi (Figure 2a), only 26.9% ± 3.7% [^99m^Tc]Tc-DB4 was found intact in peripheral mice blood in this period (Figure 2b). Co-injection of LBQ657 led to notable stabilization of the radiotracer (74.3% ± 8.6% intact; *p* < 0.0001), whereas a similar stabilization effect was observed in mice treated with Entresto 30 min prior to radioligand injection (71.5% ± 1.8% intact; *p* < 0.0001). A summary of these results in numerical values is included in Table 1.

**Figure 3:**
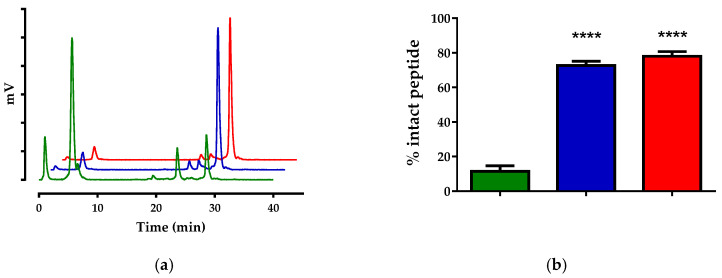
(**a**) Representative radiochromatograms of HPLC analysis (HPLC system 2b) of mice blood samples collected 5 min pi of [^111^In]In-SG4, co-injected with vehicle (green line), LBQ657 (blue line) or co-injected with vehicle 30 min after oral administration of Entresto (red line). (**b**) Comparison of percent intact [^111^In]In-SG4 across treatments (green, blue and red bars, respectively), showing statistically significant differences between LBQ657- or Entresto-treated mice vs. controls (**** *p* < 0.0001). Numerical average values ± SD are summarized in Table 1.

Likewise, HPLC analysis of blood samples collected at 5 min pi of [^111^In]In-SG4 (Figure 3a) revealed only 11.5% ± 3.2% intact radiotracer (Figure 3b).

Co-injection of LBQ657 led to notable stabilization of [^111^In]In-SG4 (72.7% ± 2.4% intact; *p* < 0.0001), with Entresto treatment inducing similar stabilization effects (78.0% ± 2.7% intact; *p* < 0.0001). A summary of these results in numerical values is also included in Table 1.

#### 3.2.2. Biodistribution of [^99m^Tc]Tc-DB4 SCID Mice Bearing PC-3 Xenografts

The biodistribution of [^99m^Tc]Tc-DB4 at 4 h pi was studied in SCID mice bearing subcutaneous PC-3 tumors expressing the human GRPR, without or during treatment with LBQ657 or Entresto. Biodistribution data, expressed as mean %IA/g ± SD, *n* = 4, is summarized in Table 2. The radiotracer has washed out from the blood and the body of mice predominantly via the kidneys and the urinary system, with some degree of intestinal uptake. High uptake is seen in the GRPR-rich pancreas (27.71 ± 6.36%IA/g) as well as in the implanted tumors (7.13 ± 1.76%IA/g).

Co-injection of LBQ657 or treatment of mice with Entresto resulted in clear enhancement of tumor uptake (to 16.17 ± 0.71%IA/g; *p* < 0.0001 and to 17.50 ± 3.70%IA/g; *p* < 0.0001 respectively, vs. controls). No significant increase was observed in any other organ or tissue, except for the pancreas. In this case, the radioligand stabilization by either LBQ657 or Entresto led to significant increases of pancreatic uptake in the mice (to 56.06 ± 4.24%IA/g; *p* < 0.0001 and to 60.98 ± 6.41%IA/g; *p* < 0.0001 respectively, vs. controls). It should be noted that uptake in the tumors as well as in pancreas and in mice intestines was significantly reduced in mice co-injected with excess [Tyr^4^]BBN 30 min after treatment with Entresto, revealing a GRPR-mediated process (to 0.55 ± 0.04%IA/g; *p* < 0.0001, to 0.68 ± 0.04%IA/g; *p* < 0.0001 and to 1.23 ± 0.21%IA/g; *p* < 0.01 respectively, vs. the Entresto group).

#### 3.2.3. Biodistribution of [^111^In]In-SG4 in SCID Mice Bearing Twin A431-CCK_2_R((+/−)) Xenografts

The biodistribution of [^111^In]In-SG4 at 4 h pi was studied in SCID mice bearing subcutaneous double A431-CCK_2_R((+/−)) tumors, without or during treatment with LBQ657 or Entresto. Biodistribution data is summarized in Table 3, expressed as mean %IA/g ± SD, *n* = 4.

The uptake of [^111^In]In-SG4 significantly increased only in the A431-CCK_2_R(+) xenografts after treatment of mice with either LBQ657 (from 3.07 ± 0.87%IA/g to 8.11 ± 1.45%IA/g; *p* < 0.0001) or Entresto (from 3.07 ± 0.87%IA/g to 9.61 ± 1.70%IA/g; *p* < 0.0001), but not in the A431-CCK_2_R(−) tumors, suggesting CCK_2_R-specificity. Non-significant changes were observed in all other tissues, except for the CCK_2_R-positive stomach, showing higher uptake during treatment with LBQ657 (from 1.29 ± 0.25%IA/g to 3.10 ± 0.41%IA/g; *p* < 0.001) or Entresto (from 1.29 ± 0.25%IA/g to 2.86 ± 0.53%IA/g; *p* < 0.01).

**Figure 4:**
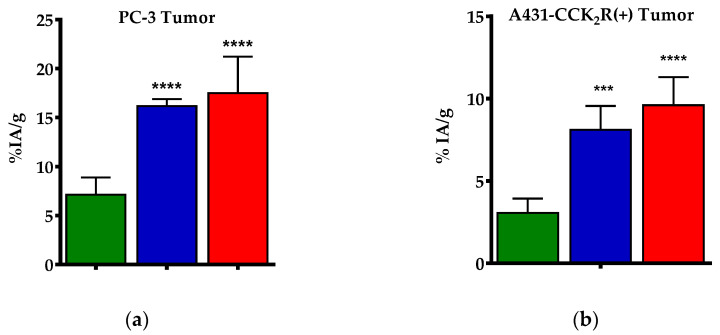
Comparative uptake at 4 h pi (as mean %IA/g values ± SD, *n*= 4) of (**a**) [^99m^Tc]Tc-DB4 in PC-3 GRPR-expressing xenografts without (green bars) or during treatment of mice with either LBQ657 (blue bars) or Entresto (red bars), and (**b**) [^111^In]In-SG4 in A431-CCK_2_R(+) xenografts without (green bars) or during treatment of mice with either LBQ657 (blue bars) or Entresto (red bars). Statistically significant differences were observed for both radioligands between mice treated with either NEP-inhibitor vs. controls (*** *p* < 0.001, **** *p* < 0.0001), but not between the LBQ657 and Entresto groups (*p* > 0.05).

Cumulative data on the impact of LBQ657 and Entresto on the uptake of [^99m^Tc]Tc-DB4 and [^111^In]In-SG4 on PC-3 and A431-CCK_2_R(+) xenografts respectively, is presented in Figure 4.

#### 3.2.4. SPECT/CT of [^111^In]In-SG4 in A431-CCK_2_R(+) Xenograft-Bearing Mice

**Figure 5:**
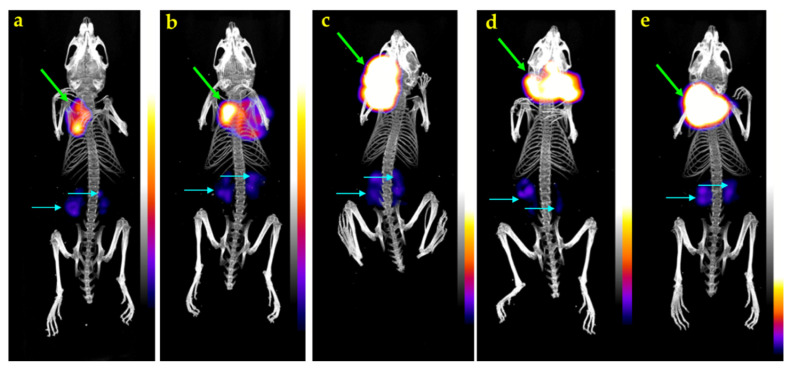
Static whole-body SPECT/CT images of five SCID mice bearing A431-CCK_2_R(+) tumors 4 h after injection of [^111^In]In-SG4 (**a**,**b**) without, or (**c**–**e**) with pre-treatment with Entresto, received orally 30 min in advance. Intense uptake is seen on tumor (green arrows), with kidneys showing only weakly in comparison (turquois arrows). A notable increase in the tumor uptake has resulted after treatment of mice with Entresto (**c**–**e**) versus controls (**a**,**b**). The color bar indicates the difference in accumulated activity (purple being the lowest and white the highest level of accumulation).

Mice SPECT/CT images obtained 4 h after injection of [^111^In]In-SG4 are presented in Figure 5. Significant accumulation was achieved in the CCK_2_R-expressing A431-CCK_2_R(+) xenografts, while much lower radioactivity levels remained in the kidneys. Clear differences in tumor uptake could be observed between controls (Figure 5a,b) and Entresto-treated mice (Figure 5c–e), in line with biodistribution findings.

## 4. Discussion

A major challenge in a wider application of peptide radioligands in cancer theranostics is linked to their fast catabolism in the biological milieu by peptidases [10,11]. We have previously reported on the prominent role of NEP in the rapid in vivo degradation of radioligands originating from various peptide families, including bombesin and gastrin [12]. NEP is an ectoenzyme with a broad substrate repertoire anchored on epithelial cell membranes of blood vessels and major organs of the body in high local concentrations [13,14]. A great number of radiopeptide analogs entering the circulation become exposed to its action and get swiftly degraded, with only a fraction of the original number of molecules reaching the tumor-associated receptors intact. As a result, diagnostic contrast and/or therapeutic efficacy are strongly compromised. We were able to successfully interfere with this chain of disadvantageous events by inducing transient NEP-inhibition through co-injection of PA [15,16] in mice. As a result, marked increases in the metabolic stability of various circulating peptide radiotracers could be achieved, directly favoring tumor uptake [12,17,18,19,20,21,24]. These exciting results, documented at the preclinical level in a multitude of cases, warrant further validation in cancer patients. Clinical proof of this concept may promptly enrich the arsenal of anti-cancer theranostic tools, since the profiles of available radiopeptides can be optimized in situ. In this way, time-/cost-intensive structure–activity relationship studies with the development of new radiotracer libraries may be circumvented.

Toward this goal, we have decided to explore the efficacy of approved NEP-inhibitors that could replace PA in a clinical setting [21,25]. Most interestingly, Entresto was approved and became commercially available recently as an antihypertensive drug [26,27,28]. Entresto pills for oral administration contain a combination of valsartan and sacubitril. The latter is the precursor form of the potent NEP-inhibitor sacubitrilat (LBQ657) [29], which is quickly released in vivo via de-esterification by endogenous esterases (Figure 1c). In the present work, we were interested to study the effects of both Entresto and the active substance LBQ657 on the stability and biodistribution profile of two radiopeptide examples, [^99m^Tc]Tc-DB4 and [^111^In]In-SG4 (Figure 1a, b, respectively). LBQ657 was intravenously injected in mice together with either radioligand. The pill was orally administered 30 min in advance of radiotracer injection, so as to accomplish similar levels of the active substance in mice plasma and hence similar NEP-inhibition efficacy [32].

As depicted in Figure 2 and Figure 3, neither of the two radiotracers were detected intact in peripheral mice blood in the absence of inhibitors as soon as 5 min pi, revealing a very rapid action of native peptidases. Co-injection of the NEP-inhibitor significantly increased the metabolic stability of both radiopeptides, directly implicating NEP in their swift in vivo degradation. Most interestingly, treatment of mice with the pill induced the same stabilization levels of the radiotracers, confirming the indistinguishable efficacy of Entresto and LBQ657 to inhibit NEP in situ [29,32]. It should be noted that PA was previously shown to stabilize BBN-/MG-based radiotracers, including [^111^In]In-SG4, with comparable efficacy [12].

We next investigated how the aforementioned radioligand stabilization affected tumor targeting and overall pharmacokinetics of the two radioligands. As shown in Table 2, we could observe a highly significant increase in the uptake of [^99m^Tc]Tc-DB4 in the PC-3 xenografts at 4 h pi in both Entresto- and LBQ657-treated mice compared with untreated controls, with the two treatments being equally effective. It should be noted that in the “block” animal group, whereby mice were treated with Entresto and a high excess of [Tyr^4^]BBN, tumor uptake was minimized. This finding is suggestive of GRPR-specificity, ruling out GRPR-unrelated interferences of the pill in the observed tumor uptake enhancement. Additional increases of radioactivity levels could be observed in mice pancreas and intestines. Such increases could be attributed to the known physiological expression of GRPR in the healthy organs, as implied by the respective values in the “block” animal group. Again, similar observations were previously reported for most BBN-based radioagonists after treatment of mice with PA [12,17,18,19,35].

The impact of treating mice with Entresto and LBQ657 on the uptake of [^111^In]In-SG4 in the A431-CCK_2_R(+) xenografts compared with untreated controls is summarized in Table 3. Again, the stabilization of the radiotracer in peripheral mice blood by either Entresto or LBQ657 led to significant and comparable increases in the CCK_2_R-expressing tumors compared with untreated controls. Furthermore, the observed enhancements were apparent only in the A431-CCK_2_R(+), but not in the A431-CCK_2_R(−) xenografts, suggesting CCK_2_R-specificity. Similar enhancements could be induced previously by transient NEP-inhibition by PA [12,20,21,24]. In view of the fact that background radioactivity levels remained the same between the Entresto and the control animal groups, including the mice CCK_2_R-rich gastric mucosa, we further proceeded to visualize these effects by SPECT/CT. As shown in the representative images of Figure 5 taken 4 h pi of [^111^In]In-SG4, a striking increase of tumor uptake could be established in the Entresto-treated mice group over the non-treated controls.

## 5. Conclusions

This study has shown that Entresto, an approved antihypertensive pill to be taken orally and promptly releasing the potent NEP-inhibitor LBQ657 in vivo [29], can induce comparable stabilization effects with LBQ657 and the non-licensed NEP-inhibitor PA of GRPR-/CCK_2_R-directed radiopeptides. The observed stabilization was shown to drastically improve the uptake of two representative radiopeptides, [^99m^Tc]Tc-DB4 and [^111^In]In-SG4, in tumor-bearing mice via GRPR-/CCK_2_R-specific pathways, most probably by enhancing the supply of intact radiotracers to tumor-residing receptors. These findings represent an important further step for the proof-of-principle of this approach in cancer patients and may thus contribute in a broader and more effective application of radiopeptides in nuclear oncology.

## Figures and Tables

**Table 1:** Stabilities of [^99m^Tc]Tc-DB4 and [^111^In]In-SG4 in peripheral mouse blood 5 min pi.

Treatment	[^99m^Tc]Tc-DB4	[^111^In]In-SG4
Control	26.9 ± 3.7 (*n* = 3)	11.5 ± 3.2 (*n* = 4)
LBQ	74.4 ± 8.6 (*n* = 3)	72.7 ± 2.4 (*n* = 3)
Entresto	71.5 ± 1.8 (*n* = 4)	78.0 ± 2.7 (*n* = 3)

Data represents the mean percentage of intact radioligand ± SD; *n* of experiments are shown in parentheses.

**Table 2:** Biodistribution data for [^99m^Tc]Tc-DB4, expressed as %IA/g mean ± SD, *n* = 4, in PC-3 xenograft-bearing SCID mice at 4 h pi without or during treatment with either LBQ657 or Entresto.

Tissue	[^99m^Tc]Tc-DB4—4 h pi			
	Controls ^1^	LBQ657 ^2^	Entresto ^3^	Block ^4^
Blood	0.12 ± 0.11	0.14 ± 0.03	0.17 ± 0.03	0.08 ± 0.01
Liver	1.76 ± 0.74	1.61 ± 0.19	1.27 ± 0.20	0.90 ± 0.10
Heart	0.24 ± 0.13	0.26 ± 0.03	0.23 ± 0.07	0.13 ± 0.00
Kidneys	9.56 ± 4.35	4.67 ± 1.03	7.86 ± 2.46	4.72 ± 0.72
Stomach	1.50 ± 0.71	1.12 ± 0.20	1.66 ± 0.30	0.20 ± 0.04
Intestines	7.99 ± 0.08	9.00 ± 0.64	11.36 ± 0.95	1.23 ± 0.21
Spleen	1.92 ± 0.75	2.46 ± 0.95	1.93 ± 0.44	2.60 ± 0.48
Muscle	0.06 ± 0.02	0.06 ± 0.01	0.10 ± 0.09	0.03 ± 0.00
Lungs	0.40 ± 0.21	0.52 ± 0.18	0.66 ± 0.11	0.72 ± 0.26
Pancreas	27.71 ± 6.36	56.06 ± 4.24	60.98 ± 6.41	0.68 ± 0.04
Tumor	7.13 ± 1.76	16.17 ± 0.71	17.50 ± 3.70	0.55 ± 0.04

All animals were injected with 200 kBq/10 pmol peptide. ^1^ Mice co-injected with vehicle, ^2^ mice co-injected with LBQ657, ^3^ mice co-injected with vehicle 30 min after oral administration of Entresto and ^4^ mice co-injected with [Tyr^4^]BBN 30 min after oral administration of Entresto for in vivo GRPR-blockade.

**Table 3:** Biodistribution data for [^111^In]In-SG4 at 4 h pi, expressed as %IA/g mean ± SD, *n* = 4, in SCID mice bearing double A431-CCK_2_R(+/−) xenografts without or during treatment with either LBQ657 or Entresto.

Tissue	[^111^In]In-SG4—4 h pi		
	Controls ^1^	LBQ657 ^2^	Entresto ^3^
Blood	0.03 ± 0.02	0.07 ± 0.01	0.06 ± 0.01
Liver	0.15 ± 0.02	0.24 ± 0.02	0.23 ± 0.03
Heart	0.05 ± 0.02	0.07 ± 0.01	0.10 ± 0.01
Kidneys	2.10 ± 0.49	2.39 ± 0.11	2.40 ± 0.61
Stomach	1.29 ± 0.25	3.10 ± 0.41	2.86 ± 0.53
Intestines	0.46 ± 0.05	0.49 ± 0.12	0.34 ± 0.07
Spleen	0.11 ± 0.04	0.23 ± 0.06	0.22 ± 0.05
Muscle	0.03 ± 0.02	0.07 ± 0.02	0.05 ± 0.01
Lungs	0.06 ± 0.01	0.10 ± 0.01	0.11 ± 0.01
Pancreas	0.08 ± 0.09	0.31 ± 0.01	0.34 ± 0.14
Femur	0.10 ± 0.03	0.13 ± 0.01	0.14 ± 0.03
Tumor (+)	3.07 ± 0.87	8.11 ± 1.45	9.61 ± 1.70
Tumor (–)	0.28 ± 0.21	0.28 ± 0.01	0.40 ± 0.23

All animals were injected with 50 kBq/10 pmol peptide. ^1^ Mice co-injected with vehicle, ^2^ mice co-injected with LBQ657 and ^3^ mice co-injected with vehicle 30 min after oral administration of Entresto.
